# TiO_2_/CuO/Cu_2_O Photovoltaic Nanostructures Prepared by DC Reactive Magnetron Sputtering

**DOI:** 10.3390/nano12081328

**Published:** 2022-04-12

**Authors:** Grzegorz Wisz, Paulina Sawicka-Chudy, Maciej Sibiński, Dariusz Płoch, Mariusz Bester, Marian Cholewa, Janusz Woźny, Rostyslav Yavorskyi, Lyubomyr Nykyruy, Marta Ruszała

**Affiliations:** 1 Institute of Materials Engineering, College of Natural Science, University of Rzeszow, 1 Pigonia Street, 35-959 Rzeszow, Poland; gwisz@ur.edu.pl (G.W.); dploch@ur.edu.pl (D.P.); mruszala@ur.edu.pl (M.R.); 2 Department of Semiconductor and Optoelectronic Devices, Lodz University of Technology, Wólczańska 211/215, 90-924 Lodz, Poland; maciej.sibinski@p.lodz.pl (M.S.); janusz.wozny@p.lodz.pl (J.W.); 3 Institute of Physics, College of Natural Science, University of Rzeszow, 1 Pigonia Street, 35-317 Rzeszow, Poland; mbester@ur.edu.pl (M.B.); mcholewa@ur.edu.pl (M.C.); 4 Department of Physics and Chemistry of Solids, Vasyl Stefanyk Precarpathian National University, T. Shevchenko, 57, 76-018 Ivano Frankivsk, Ukraine; roctyslaw@gmail.com (R.Y.); lyubomyr.nykyruy@pnu.edu.ua (L.N.)

**Keywords:** solar cells, copper oxide, titanium dioxide, reactive magnetron sputtering

## Abstract

In this study, titanium dioxide/copper oxide thin-film solar cells were prepared using the reactive direct-current magnetron sputtering technique. The influence of the deposition time of the top Cu contact layer on the structural and electrical properties of photovoltaic devices was analyzed. The structural and morphological characterization of the TiO_2_/CuO/Cu_2_O solar cells was fully studied using X-ray diffraction (XRD), scanning electron microscopy (SEM), and current–voltage (I-V) characteristics. Additionally, using van der Pauw sample geometries, the electrical properties of the titanium dioxide and copper oxide layers were investigated. From the XRD study, solar cells were observed in cubic (Cu_2_O), monoclinic (CuO), and Ti_3_O_5_ phases. In addition, the crystallite size and dislocation density for copper oxide layers were calculated. Basic morphological parameters (thickness, mechanism of growth, and composition of elements) were analyzed via scanning electron microscopy. The thicknesses of the titanium dioxide and copper oxide layers were in the range of 43–55 nm and 806–1223 nm, respectively. Furthermore, the mechanism of growth and the basic composition of the elements of layers were analyzed. The I-V characteristic curve confirms the photovoltaic behavior of two titanium dioxide/copper oxide thin-film structures. The values of short-circuit current density (J_sc_) and open-circuit voltage (V_oc_) of the solar cells were: 4.0 ± 0.8 µA/cm^2^, 16.0 ± 4.8 mV and 0.43 ± 0.61 µA/cm^2^, 0.54 ± 0.31 mV, respectively. In addition, the authors presented the values of I_sc_, P_max_, FF, and R_sh_. Finally, the resistivity, carrier concentration, and mobility are reported for selected layers with values reflecting the current literature.

## 1. Introduction

In recent years, researchers have shown great interest in the analysis of thin-film solar cells or dye-sensitized photovoltaics [1,2,3,4,5]. A main advantage of these photovoltaic devices is their lower panel cost. Further, layers of thin film solar cells could make deposition on flexible substrates and integration with other objects possible [3].

Copper oxide and titanium dioxide are interesting semiconductors for photovoltaic applications because of their physical properties. They are non-toxic, abundant, and inexpensive [6,7]. Copper oxide can have one of two forms known as p-type metal oxide semiconductors (MO): copper oxide (CuO) and cupric oxide (Cu_2_O) [8]. On the other hand, stoichiometric titanium dioxide (IV) dioxide has many suboxides (e.g., Ti_3_O_5_, Ti_4_O_7_, or Ti_8_O_15_). Ti_3_O_5_ could be applied as a functional material in oxygen sensors or catalyst applications, but not as a layer in thin film solar cells [9].

Several studies have described titanium dioxide/copper oxide solar cells with various techniques. For example, Siripala et al. [10] fabricated Cu_2_O/TiO_2_ structures by electrochemical deposition of copper oxide (I) on titanium foil [10]. Pavan et al. [7] prepared a TiO_2_/Cu_2_O heterojunction using spray pyrolysis [7]. Hussain et al. [11] prepared Cu_2_O/TiO_2_ solar cells using electrodeposition and radio frequency sputtered techniques [11]. In early experiments, the authors manufactured titanium dioxide/cuprous oxide solar cells [12,13]. In the current literature, 1.62% has been the highest conversion efficiency achieved for titanium oxide and copper oxide-based photovoltaic devices [14]. However, theoretical simulations are very promising; 13.7% for TiO_2_/Cu_2_O [15,16] and 19.65% for TiO_2_/CuO [17].

Thin-film solar cells were prepared using reactive direct-current magnetron sputtering (DC-MS). Their details are described in [18]. In this article, the authors present a continuation of their analysis. The sample with the highest efficiency was compared with two other samples prepared with an extended deposition time of the Cu top contact and a very similar time of deposition of TiO_2_ layers. In comparison to previous published papers [12,13,18], in this manuscript the authors study the influence of the deposition time of the top Cu contact layer on the structural and electrical properties of TiO_2_/CuO/Cu_2_O solar cells. The analyses confirmed that a proficient understanding of technological processes plays a key role, leading to the efficient creation of heterostructures and a proper contact system.

## 2. Materials and Methods

Thin films for solar cells were grown using a Ti target (99.995%) for TiO_2_ and a Cu target (99.995%) for Cu_2_O/CuO layers with a diameter of 25.4 mm obtained from Kurt J. Lesker Company. Base pressure was below 10^−3^ Pa (10^−6^ mbar) before deposition began. Commercial glass-coated indium-tin oxide (ITO) and silicon were used as substrates. The area of the samples was 1.0 ± 0.1 cm^2^. The substrate temperature during the deposition of the thin films was kept at 300 °C. O_2_/Ar mixtures with a ratio of 4:1 and 4:0.5 for the TiO_2_ and CuO layers were selected as an active gas for plasma sputtering.

**Figure 1:**
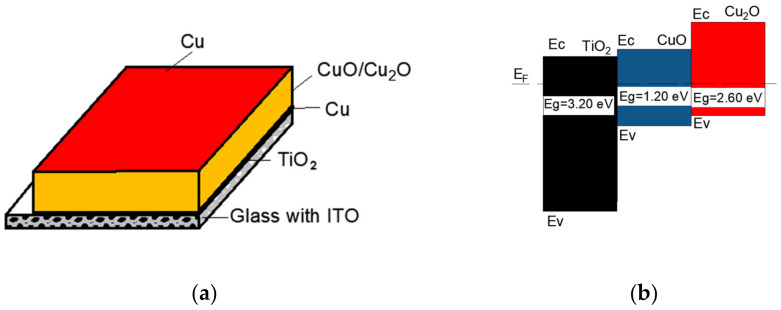
(**a**). Scheme of the layers deposited by direct-current magnetron sputtering on glass with ITO to create a thin film solar cell. (**b**). The energy level band diagram of the TiO_2_/CuO/Cu_2_O structure.

The deposition process of these layers was as follows: the titanium oxide layers (IV) were deposited on glass covered by ITO; next, a Cu buffer was deposited for 5 s for each sample; then, copper oxide was grown on top of the Cu buffer; finally, a thin Cu film was deposited on the top of the copper oxide as a contact for 20 s for #12 and 60 s for #14 and #15 (Figure 1a). Details of the sputtering conditions are listed in Table 1. The different process parameters are marked in bold. The process parameters and results of sample #12 were obtained with permission from G. Wisz et al. [18].

The schematic energy level band diagram of the TiO_2_/CuO/Cu_2_O, based on Refs. [13,16,19,20,21], is shown in Figure 1b, where: Eg are the energy band gaps of TiO_2_, CuO, and Cu_2_O, E_F_ is Fermi level, and Ev and Ec are valence and conduction bands, respectively.

For better understanding of the deposition process authors invite reader(s) to view a YouTube video of the PVD laboratory and the PREVAC apparatus, which are linked in the Supplementary Materials section.

The crystal structure properties of the grown TiO_2_/Cu_2_O/CuO were investigated using X-ray diffraction (XRD) with a CuKα radiation source, a Bruker D8 Discover diffractometer. Analyses of the XRD patterns were performed using HighScore Plus and Diffrac. EVA software with an ICDD PDF-2+ crystallographic database.

SEM images of the solar cells were taken using a Helios NanoLab 650 scanning electron microscope from the FEI company. An additional EDS detector was used to characterize the chemical composition of the structures with an acceleration voltage setting value of 20 kV. The current–voltage characteristics of the photovoltaic structures were measured using an I-V meter (Keithley 2062) under illumination of ~500 W/m^2^ from a halogen lamp. Finally, electrical properties were measured using the van der Pauw method. Active layers were characterized in terms of resistivity, carrier concentration, and mobility.

## 3. Results and Discussion

### 3.1. XRD Study

**Figure 2:**
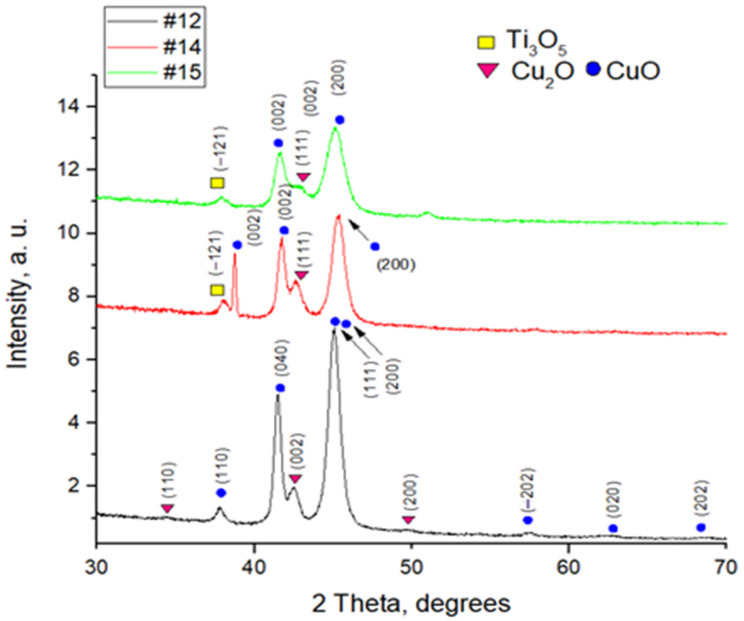
X-ray diffraction patterns of #12, #14, and #15 solar cells.

The composition characterization of the solar cells was carried out by XRD patterns in the range of 2 Theta between 30° and 70° (Figure 2).

For sample #12, two of the highest intense diffraction peaks (which indicate that the copper oxide layer is well crystallized) are observed at values 41.5°, 44.9°, and 45.3°. They can be indexed as monoclinic structured copper oxide–CuO(C2/c). Their preferred orientations are (040), (111), and (200) along the axis, respectively.

Other less intense peaks for CuO(C2/c) are obtained at 37.8°, 57.6°, 62.4°, 68,1° and correspond to (110), (−202), (020), (202) and for Cu_2_O (Pn-3m) at 34.4°, 42.5°, 49.4° corresponding to (110), (002), (200).

For samples #14 and #15, less intense peaks are obtained for monoclinic CuO at 38.7°, 41.6°, 45.3° and 41.6°, 45.3° respectively.

The XRD spectrum of samples contained no peaks for TiO_2_, most likely due to a very thick copper oxide layer.

The XRD study indicates that only the copper oxide in sample #12 is well crystallized due to intense and sharp diffraction peaks. Furthermore, the diffraction spectrum of samples #14 and #15 shows wider and lower peaks, so these layers are amorphous, which may affect the low PV parameters. Additionally, for #14 and #15, XRD patterns show the phase of Ti_3_O_5_. Ti_3_O_5_ which could be treated as a layer defect.

Using the full width at half maximum, the variation of the crystallite size formed in samples #12, #14, #15 was calculated from the most intense peak using the Debye-Scherrer formula [22] and is presented in Table 2 below.

(1) $$t=\frac{0.9\lambda }{\mathrm{FWHMcos}\theta }$$

where t is the crystallite size, FWHM is the full width at half maximum of the spectrogram curves, θ is Bragg’s angle and λ is the wavelength of the CuKα radiation (1.5406A). The results of 2 Theta, FWHM, orientation, and calculation of crystallite sizes are listed in Table 1.

The average crystallite size for CuO is 12 nm (#12), 9 nm (#14), 12 nm (#15); and for Cu_2_O 11 nm (#12), 10 nm (#14), 8 nm (#15).

Furthermore, the dislocation density (δ) has been calculated [23] using the following relations:

(2) $$\delta =\frac{1}{t^{2}},\;$$

The measured lattice parameters a, b, c, (a ≠ b ≠ c), and for CuOα = ɤ =90° ≠ β, thickness and δ are summarized in Table 3. The dislocation density is similar for samples #12 and #14 and increases for sample #15 due to the increase of the amorphous layer.

### 3.2. Structural Characterization

**Figure 3:**
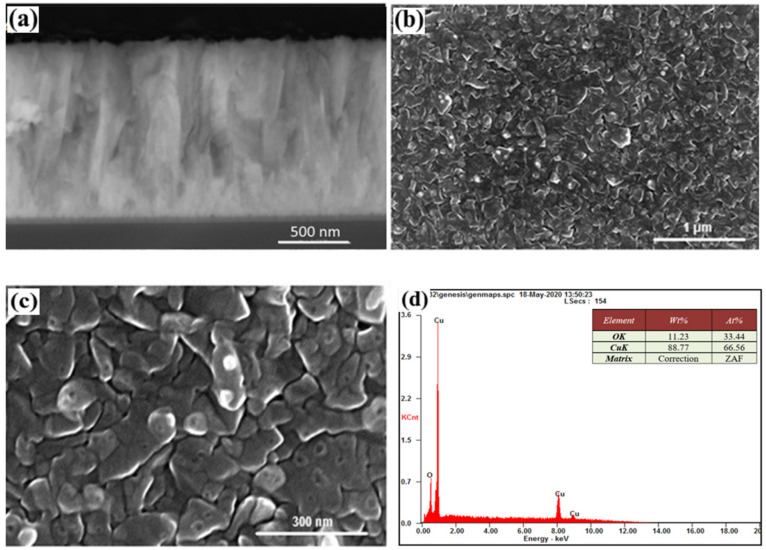
Cross-section (**a**), morphology (**b**,**c**) (for (**b**) reprinted with permission from Ref. [18]. Copyright 2021, Polish Academic of Sciences), and (**d**) chemical analysis of sample #12.

The SEM images for sample #12 show clear growth in the form of separate columns that do not coagulate with each other. The height of each column is close to the film thickness, and are clearly visible in the cross section in Figure 3a. The discrepancy in the values of the lattice parameters for TiO_2_ and Si causes the presence of significant deformation stresses of the ‘substrate film’ type, which in the initial stages of growth cause the nucleation of islands in the form o4 individual columns. The width of these columns is constant at ~80–300 nm (Figure 3c), which is consistent with the images of the cross section of the film. Moreover, in Figure 3b, homogeneity is well-observed in accordance with such columns. According to chemical analyses, the atomic content of copper (Figure 3d) was 66.56% and its oxygen content 33.44%. It can be seen from these structural analyses that the SEM image of sample #12 was uniform in color (Figure 3b) and the atomic concentration ratio between Cu and O was ~2.

**Figure 4:**
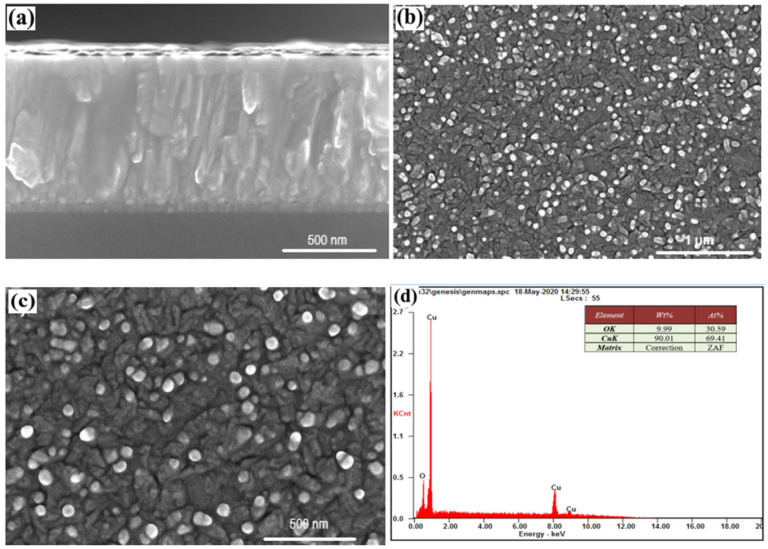
Cross-section (**a**), morphology (**b**,**c**), and chemical analysis (**d**) of sample #13.

The column type of the film growth, which is rounded at the top, can also be observed in sample #14, (Figure 4b,c). Based on the EDS analysis, we can conclude that there were no separate phases other than copper (the light objects in Figure 4b,c), the atomic percentage of which was 69.41% copper and 30.59% oxygen.

**Figure 5:**
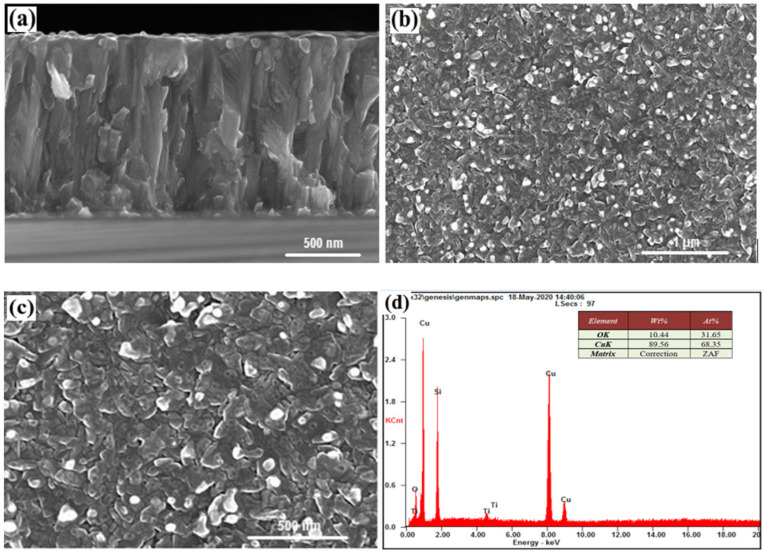
Cross-section (**a**), morphology (**b**,**c**), and chemical analysis (**d**) of sample #15.

In sample #15, the growth of the columns is seen developing in the same direction of the preferred orientation of the c-axis (Figure 5a). The observed structure resembles a continuous film with vertical chips along the direction of growth (Figure 5b,c).

Table 4 shows the basic morphological parameters of the deposited thin films.

Considering the heterostructural composition of the previous results [18], it is possible to confirm that the oriented Si determines the directional growth of TiO_2_ (column diameter evenly distributed and is ~20–25 nm; the height of the columns is ~50–60 nm). In turn, this structure of TiO_2_ determines a columnar growth type for Cu_2_O. These Cu_2_O columns expand slightly with increasing film thickness from the substrate at the top of the heterostructure. Such column-type growth is observed for layers with highly-mismatched layers between the deposited material and the substrate. High lattice mismatch between TiO_2_ and Cu_2_O leads to Volmer–Weber growth mechanism for which island growth takes place directly on the substrate without formation of the wetting layer [24,25].

The technological implementation of the column growth mechanism is significant from a practical point of view [26,27]. After all, the formation of columns of a given size allows for control over the number of boundaries between them, which are structural defects, the size and shape of which determine several physical characteristics of the material (such as effect of refraction and, accordingly, improving of absorption, material resistance, etc.) [28].

It should also be noted that the application of a Cu contact layer for Cu_2_O causes the formation of pure Cu phases on the surface (light spots in Figure 3b,c, Figure 4b,c and Figure 5b,c). Such phases are located on the surface of individual columns, the boundaries of which resemble the boundaries of the grains. The surface phases of Cu have dimensions of about 50–75 nm for all samples. They can be considered as the release of a certain phase on the surface of the film. In contrast to such phase inclusions, the columns can be considered as grainy, based on their surface structure, and physical and mathematical models can be used that describe the effect of ‘grains’ and grain boundaries on material properties [29]. The effect of the Cu layer, which has a lattice parameter value close to it in TiO_2_ (anatase) leads to the implementation of Frank–van der Merwe growth mechanism for which film growth by emergence of two-dimensional islands, or due to formation of monoatomic steps [30].

Based on SEM analyses of sample # 12, the Cu layers, which are deposited at the end of the process, were oxidized to the Cu_2_O compound. This is not observed for samples #14 and #15. Figure 3 and Figure 4 show a certain difference in morphology compared to sample #12. Regular, round-shaped individual light-surface nanostructures, observed through SEM in the form of secondary electrons, clearly indicate the presence of individual phases of heavier elements on their surfaces.

### 3.3. I-V Characteristics

**Figure 6:**
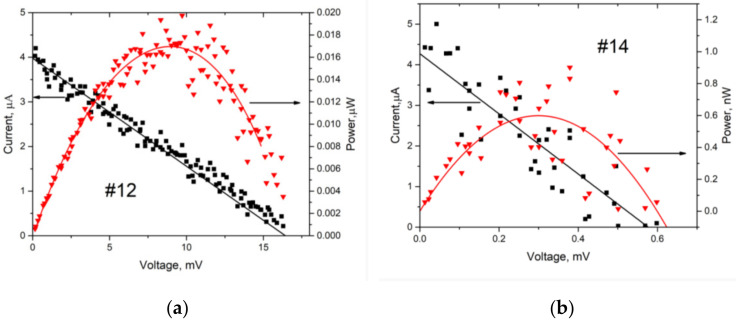
(**a**). Light I-V and P-V characteristics of heterojunctions for #12. (**b**). Light I-V and P-V characteristics of heterojunctions for #13.

Figure 6a,b show light I-V and P-V characteristics for samples #12 and #14, respectively. Considering the I-V curves for #12 and #14 of the investigated samples, this confirms the presence of a photovoltaic effect. The I_sc_, J_sc_, V_oc_, P_max_, FF, and R_sh_ obtained from solar cells are shown in Table 5. Sample #15 is not included in Table 5 due to its non-existent PV effect.

### 3.4. Active Layer Electrical Properties/Measurements

Electrical properties were measured using the van der Pauw method. Active layers were characterized in terms of resistivity, carrier concentration and mobility. Samples were prepared using the same process utilized for the completion of thin solar cells. The TiO_2_ and CuO/Cu_2_O layers were deposited separately on an oxidized Si substrate. Four contact points were located at the corners of the rectangular-shaped samples. Contact points with CuO samples were formed using a silver conductive adhesive; though, for the TiO_2_ layers, a Ti conductive adhesive was used. A Keithley2450 SMU unit was used as the current source and the voltage was measured using a Keithley 7510 voltmeter. The resistivity was obtained from van der Pauw measurements. For samples with ohmic contact points, the mobility and carrier concentration were obtained using the Hall measurement. The measurement procedure followed the steps described in [31].

**Figure 7:**
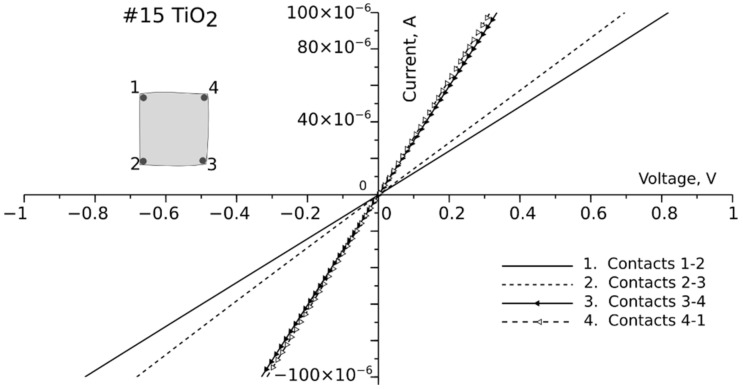
I-V characteristics of side contacts for #15 CuO and #15 TiO_2_.

To correctly measure the Hall voltage, there must be a linear contact point between the active layers and probes. The quality of these points was verified by sweeping the current when each connection configuration was in the range of <−Imax, Imax>. The selected Imax range was from 0.001 to 1.0 mA, depending on the voltage drop and the thickness of the active layer. These measurements for selected layers are shown in Table 6. High-quality contact points were obtained for samples #15 CuO and #15 TiO_2_, as shown in Figure 7. The resistivity of TiO_2_ was measured for sample #15, where ohmic contact was obtained using Ti powder conductive glue.

Pure TiO_2_ is an insulator (with a resistivity greater than 1 × 10^10^ Ω⸱cm [29]). However, when doped (with atomic dopants or in the presence of defects), its resistivity can be reduced to 10^−4^ Ω⸱cm [32,33]. The resistivity of #15 TiO_2_ was 0.013 Ω⸱cm. The measured carrier concentration of this layer was high at 9.62 × 10^19^ cm^−3^, and its mobility was 5.04 cm^2^/(Vs). Similar values were reported by Krasienapibal et al. in [34].

The resistivity of the copper oxide samples differed significantly. In sample #14 CuO, the contact points were not linear, and therefore mobility and carrier concentration could not be calculated. The highest resistivity was found for sample #12 CuO (1.57 Ω·cm), while in #15 CuO, the resistivity only reached 0.0023 Ω·cm. This low resistivity was due to the presence of the Cu + Cu_2_O layer, which has also been reported in the existing literature [35].

From measurements of the electrical properties of the layers, it can be seen that the layers are sensitive to the parameters used in the manufacturing process. The resistivity of the solar cell layers should be as low as possible [36], however, the appropriate atomic structure must be maintained to correctly form the p-n junction. Although sample #15 shows the lowest resistivity, it does not reveal the photovoltaic effect. Theoretically, the efficiency of TiO_2_/CuO could reach 20% [15]. However, this would require carrier mobilities greater than 100 cm^2^V^−1^s^−1^which are to be achieved.

## 4. Conclusions

In this study, copper oxide/titanium dioxide solar cells were prepared using reactive, direct-current magnetron sputtering. The main aim of this article was to present the effects of the deposition times of the top contact point of Cu and TiO_2_ layer on the structural and electrical properties of solar cells. Three TiO_2_/CuO/Cu_2_O solar cells within the series were characterized using X-ray diffraction, scanning electron microscopy, and current–voltage characteristics. The electrical properties of the layers were also studied.

The lack of expected efficiency in all investigated samples is evident when analyzing their I-V characteristics. Their proper curve characteristics are hindered by the low FF parameters caused by improper cell resistance values derived from unoptimized morphological structures, and possible internal short-circuits, caused by Cu diffusion.

Electrical measurements show that even a small variation in the processing parameters leads to a drastic change in the electrical properties.

Structural improvements in the manufactured junctions and the contact point system may lead to higher overall coherence in this area of study.

## Figures and Tables

**Table 1:** Deposition conditions for #12, #14, and #15.

Parameter	#12		#14		#15	
	TiO_2_	CuO/Cu_2_O	TiO_2_	CuO/Cu_2_O	TiO_2_	CuO/Cu_2_O
Interlayer Cu buffer	5 s					
Time [min]	23	25	25	25	20	25
Power [W]	120	70	120	70	120	70
Pressure process [Pa]	1.1	1.1	1.2	1.2	1.1	1.1
Distance between the source and substrate [mm]	58	58	58	58	58	58
Oxygen flow rates [cm^3^/s]	4	4	4	4	4	4
Argon flow rates [cm^3^/s]	0.5	1	0.5	1	0.5	1
Substrate temperature [°C]	300	300	300	300	300	300
Cu top contact	20 s		60 s		60 s	

**Table 2:** Bragg angle 2 Theta, FWHM, composition, crystallite sizes for copper oxide.

	#12		#14		#15	
2 Theta (°)	45.3	42.5	45.3	42.6	45.3	42.6
FWHM (rd)	0.0084	0.0134	0.0098	0.0138	0.0119	0.0192
Composition	CuO	Cu_2_O	CuO	Cu_2_O	CuO	Cu_2_O
Orientation	(200)	(002)	(200)	(111)	(200)	(111)
Crystallite sizes [nm]	12	11	9	10	12	8

**Table 3:** Structural parameters of TiO_2_/CuO/Cu_2_O thin films.

	Phases	Thickness of Layers (nm)	Lattice Parameters				δ
			a	b	c	β	×10^12^ (m^−2^)
			(Å)			(°)	
#12	CuO	1223 ± 5	4.265(8)			98.82(8)	4.01
	Cu_2_O		4.660(5)	3.41(1)	5.13(2)		8.12
#14	CuO	982 ± 5	4.66(1)	3.44(1)	5.11(2)	98.93(2)	12.77
	Cu_2_O		4.289(2)				8.57
#15	CuO		4.71(1)	3.48(1)	5.14(2)	98.36(1)	19.01
	Cu_2_O	806 ± 5	4.289(2)				16.67

**Table 4:** The basic morphological parameters of deposited thin films.

Sample Number	Thickness of Layers: Copper Oxide: Titanium Dioxide [nm]	Mechanism of Growth	Composition of the Elements, At%
#12	1223 ± 5:47 ± 2	Frank van der Merwe	CuK: 67 ± 1 OK: 33 ± 1
#14	996 ± 5:55 ± 2	Volmer-Weber	CuK: 69 ± 1 OK: 31 ± 1
#15	812 ± 5:43 ± 2	Volmer-Weber	CuK: 68 ± 1 OK: 31 ± 1

**Table 5:** The results of solar cells based on copper oxide and titanium dioxide.

No.	I_sc_ [µA]	J_sc_ [µA/cm^2^]	V_oc_ [mV]	P_max_ [µW]	F [%]	Rsh [Ώ]
#12	4.0 ± 0.2	4.2 ± 0.8	16.1 ± 4.8	0.017 ± 0.01	30 ± 1	4250 ± 8
#14	4.3 ± 0.2	4.3 ± 0.8	0.54 ± 0.31	0.0006 ± 0.0003	31 ± 1	1269 ± 3

**Table 6:** Resistivity of measured layers, mobility, and concentration for selected layers.

Sample #	Thickness [nm]	Resistivity [Ω·cm]	Mobility [cm^2^/(Vs)]	Carrier Concentration [1/cm^3^]
#12 CuO	1223	1.57	4.34	5.8 × 10^17^
#14 CuO	996	0.28		
#15 CuO	812	0.0023	40.3	6.77 × 10^19^
#12 TiO_2_	47	n.a. (bad contact quality)		
#14 TiO_2_	56	n.a. (bad contact quality)		
#15 TiO_2_	44	0.013	5.04	9.62 × 10^19^

## Data Availability

Data is contained within the article.

## Supplementary Materials

Link I: https://www.youtube.com/watch?v=-0Sn4UbiKaE (accessed on 10 April 2022); Link II: https://www.youtube.com/watch?v=Lavsm1CIqhY (accessed on 10 April 2022); Link III: https://www.youtube.com/watch?v=iei5bn2UAzg&t=35s (accessed on 10 April 2022); Link IV: https://www.youtube.com/watch?v=0TjWJwxLZYk&t=4s (accessed on 10 April 2022); Description: Video of our study of a thin-film solar cell based on titanium dioxide and copper oxide and demonstration of the PVD laboratory and the PREVAC apparatus at the University of Rzeszow, Poland.
